# Incidence and risk factors of refeeding syndrome in inpatients undergoing enteral and parenteral nutrition hospitalized in a university medical hospital

**DOI:** 10.1590/1414-431X2026e15114

**Published:** 2026-08-17

**Authors:** R.O. da Rocha, M.S. de Rezende, H.G. Oliveira, L.S.N. Chini, J.P. Strogoff-de-Matos

**Affiliations:** 1 Departamento de Medicina Clínica, Faculdade de Medicina, Universidade Federal Fluminense, Niterói, RJ, Brasil; 2 Hospital Universitário Antônio Pedro, Universidade Federal Fluminense, Niterói, RJ, Brasil

**Keywords:** Refeeding syndrome, Incidence, Risk factors, Enteral nutrition, Parenteral nutrition

## Abstract

Refeeding syndrome (RS) encompasses electrolyte and metabolic disturbances, and its incidence depends on diagnostic criteria. We aimed to evaluate the incidence and risk factors for RS in inpatients at a university hospital in Brazil, who received enteral or parenteral nutrition. This retrospective study included all patients admitted to the hospital who received enteral or parenteral feeding after at least a 3-day fasting period. RS diagnosis was based on phosphorus, potassium, and magnesium changes in the first 3 days after diet reintroduction. Clinical, anthropometric, and other laboratory evaluations, as well as caloric prescriptions, were also collected. Risk factors for RS development were evaluated using logistic regression, and the 30-day mortality risk associated with RS was analyzed using Cox regression. A total of 315 participants were included in the final analysis. The median age was 65 years, 55% were men, 45% had a diagnosis of cancer, and the body mass index was 23.7 (IQR: 20.5-27.0) kg/m^2^. Development of RS was found in 36.8%. The risks associated with RS development were female gender and higher serum phosphorus and sodium levels. The 30-day survival rates for patients with or without RS were 50.7 and 49.9%, respectively (P=0.42). In the Cox regression model, the diagnosis of RS was not associated with death risk (HR: 0.81, 95%CI: 0.54-1.21). In conclusion, a high incidence of RS was found, but this was not associated with increased death risk, which could be explained by the prompt diagnosis and correction of electrolyte deficits.

## Introduction

In recent decades, the use of nutritional therapy has become a common practice for hospitalized patients to avoid the deleterious effects of prolonged fasting or to treat pre-existing malnutrition. Concurrently, the risk of refeeding syndrome (RS) has been highlighted by health teams. RS can be described as a deviation of fluids and electrolytes that can occur with the reintroduction of oral, enteral, or parenteral nutrition in malnourished patients (1,2). The pathophysiological mechanism is based on the transition from a catabolic state, where fats and proteins serve as the primary energy sources, to an anabolic state, where carbohydrates become the primary source of energy. The stimulation of insulin production by carbohydrates results in an increase in glycolysis, the resumption of glycogen synthesis, fats, and proteins, and an increase in the accumulation of extracellular water and sodium.

This anabolic process requires cofactors such as thiamine (vitamin B1) and minerals like phosphorus, potassium, and magnesium, which move into the cells, rapidly lowering their serum levels. Insulin action on distal nephrons reduces the elimination of sodium and water through urine, causing rapid water retention (3).

The clinical manifestations of RS result from hypophosphatemia, hypokalemia, hypomagnesemia, thiamine consumption, and fluid overload, which can be nonspecific or lead to heart failure, arrhythmias, and respiratory failure (2). When not recognized or diagnosed, RS can be potentially fatal, as first seen in starving prisoners during World War II when they were re-fed (4). In the 1980s, patients receiving high caloric loads through parenteral nutrition presented a similar clinical picture and outcome (5).

The absence of a universally accepted definition of RS leads to highly variable incidence, depending on the patient group analyzed (6). Some current definitions are based on electrolyte disturbances, primarily hypophosphatemia, while others combine clinical signs and symptoms, such as edema, respiratory or cardiac failure, and electrolyte changes (7,8). A recent systematic review of 35 studies found that the incidence of RS ranged from 0 to 62%, clearly associated with the different definitions used and the characteristics of the participants (9). In a study involving only critically ill patients, the incidence was 34% (10), while in another with a heterogeneous hospital population, the incidence was only 1% (11). It is worth noting that many health professionals still do not recognize or diagnose RS, which may mask its true incidence and hinder its treatment (12).

The criteria of the National Institute for Health and Care Excellence (NICE) (13), the American Society of Parenteral and Enteral Nutrition (ASPEN) (2), and Friedli et al. (14) were developed to detect patients at risk of developing malnutrition. All these criteria include body mass index (BMI), percentage of weight loss, reduction in food intake, low levels of P, K, and Mg, history of alcohol or drug use, or even loss of subcutaneous fat and muscle mass. The NICE criteria demonstrated low sensitivity and specificity (15,16). The absence or unreliability of collected data, along with the need for complementary examinations, hinders the use of these tools.

Starvation is the most important predictor of RS (11,17). Other risk factors include critically ill patients, geriatric patients, especially the frail, cancer patients, patients with malabsorption syndromes, post-bariatric surgery patients, chronic alcoholics, those with short bowel syndrome, inflammatory bowel diseases, and anorexia nervosa (18).

This study aimed to estimate the incidence of RS and the risk factors most associated with its development in adult patients hospitalized at the Antônio Pedro University Hospital from 2018 to 2023 who received enteral or parenteral nutrition and its association with 30-day mortality, monitored by the hospital's multidisciplinary nutritional therapy team (MNTT).

## Material and Methods

This was a retrospective observational study of a longitudinal cohort, involving patients hospitalized in clinical and surgical wards, intensive care unit (ICU), and Coronary Unit of Hospital Antônio Pedro, Universidade Federal Fluminense Hospital, Niterói, Rio de Janeiro, Brazil, from April 2018 to June 2023. All patients over 18 years of age who received enteral or parenteral nutrition for 3 consecutive days were eligible. Pregnant women, patients with pre-existing electrolyte disorders, those on hemodialysis, in diabetic ketoacidosis, post-parathyroidectomy, end-of-life patients, and those who were prescribed oral nutritional supplements were excluded. Data were collected from the archived records of the Enteral and Parenteral Nutritional Therapy Program and from medical records. The project was approved by the Research Ethics Committee of the Department of Medicine of the Universidade Federal Fluminense (number 6.340.256).

The variables analyzed were age, gender, primary and secondary diagnoses, comorbidities, anthropometric measurements (current, usual, and adjusted weight, and BMI), type of nutritional therapy (enteral and/or parenteral), caloric and protein intake in the first three days of nutritional therapy, intravenous electrolyte repletion, and laboratory tests: sodium, chlorine, potassium, magnesium, phosphorus, calcium, serum albumin, hematocrit, hemoglobin, blood urea nitrogen (BUN), and creatinine. Serum levels of phosphorus, potassium, and magnesium were collected from day 0, before the start of nutritional therapy, and daily until day 3. Day 0 values were considered as those that were obtained up to two days before the start of nutritional support. The reference values for these electrolytes in adults by the institution's laboratory were: phosphorus, 2.5 to 4.7 mg/dL; magnesium, 1.6 to 2.6 mg/dL; and potassium, 3.5 to 4.7 mEq/L. Patients diagnosed with refeeding syndrome (14) were considered to have at least one change in electrolyte levels within 3 days after the start of nutritional therapy, including: 1) a ≥30% reduction in phosphorus from baseline level or an absolute value below 2.0 mg/dL; 2) or a drop below the normal range in at least 2 of the following electrolytes: Mg (<1.6 mg/dL), P (<2.5 mg/dL), or K (<3.5 mEq/L).

After calculating basal energy expenditure, the initial refeeding plan was to start with 1/3 of the planned value. If the patient showed clinical signs of malnutrition and had a BMI<18.5 kg/m^2^ and was considered at risk for developing refeeding syndrome, the initial caloric quota for enteral or parenteral diet was set between 5 and 15 kcal/kg/day. This amount could be maintained, increased, or suspended depending on the patient's clinical and electrolyte evolution. If refeeding began with 1/3 of the planned caloric value and clinical and electrolyte progression was unfavorable, the quota was reduced by 50%. All patients received the basic daily quota of multivitamins. Deficiencies of P, K, and Mg were corrected according to the protocols of the inpatient units. The fluid quota was managed by the attending physicians, with a suggested range of 20 to 30 mL/kg/day.

### Statistical analysis

Continuous variables are reported as means±SD for normally distributed data or as median and interquartile range otherwise. Categorical variables are reported as frequencies. Comparisons of continuous variables between groups were performed using the Student's *t*-test or Mann-Whitney U test. Comparisons among three or more groups were performed using one-way ANOVA or Kruskal-Wallis, followed by the Bonferroni test. For comparisons of proportions, the chi-squared test or Fisher's exact test was used. Logistic regression was used to test the association between independent variables and the occurrence of RS. The Kaplan-Meier method was used to analyze survival rates, and the log-rank test was used to compare the curves. RS as a predictor of death risk was analyzed using the Cox regression model adjusted for other covariates. P values <0.05 were considered significant.

The statistical analysis was performed using the SPSS program, version 18.0 for Windows (IBM, USA).

## Results

**Figure 1:**
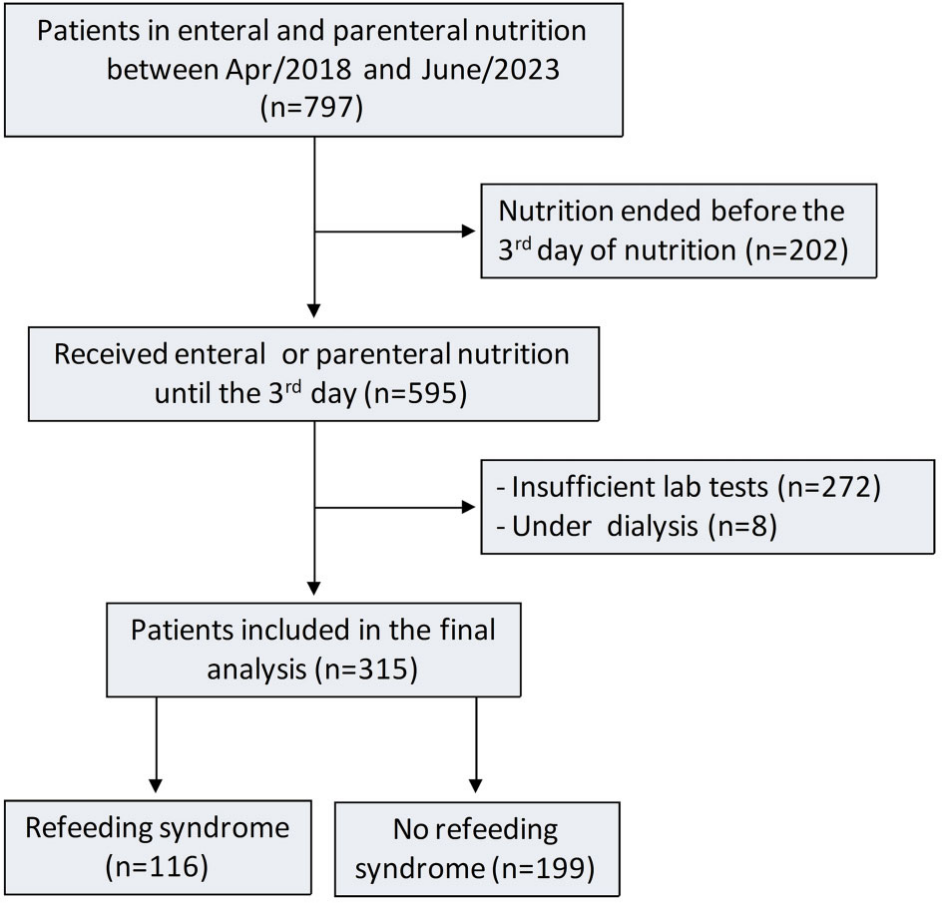
Flowchart of participant selection of the study.

Between April 2018 and June 2023, 797 patients received enteral or parenteral nutrition at the Antônio Pedro University Hospital. Of these, 595 completed the nutrition by the third day. A total of 272 were excluded due to insufficient laboratory data, and 8 were excluded for being on dialysis. Thus, 315 patients were included in the final analysis (Figure 1). According to the diagnostic criteria adopted, 116 (36.8%) developed RS.

The baseline characteristics of the sample and the comparisons between groups at the beginning of the study are described in Table 1. The sample included more males, with an average age of 65 years, average weight of 62 kg, and BMI of 23.7 kg/m^2^, with a large percentage of neoplastic patients (44.8%), most of whom had gastrointestinal (38%) and hematological (39%) cancers. More than half of the admissions were to the ICU (58.7%), followed by clinical and surgical units (28.3%). All patients had low hemoglobin (9.4 g/dL) and albumin levels (2.6 g/dL). Urea, creatinine, sodium, chloride, potassium, and magnesium values were within normal laboratory ranges. None of these variables were significantly different between the groups with and without RS. The average phosphorus level was 3.4 mg/dL (IQR: 2.6-4.5), with a significant difference between the two groups (P=0.001).

**Table 1:** Main characteristics of patients at baseline.

	Overall	No RS	RS	P-value
	(n=315)	(n=199)	(n=116)	
Female, n (%)	141 (44.8)	82 (41.2)	59 (50.9)	0.10
Age (years)	65 (56-73)	65 (57-72)	64 (54-73)	0.69
Weight (kg)	62.9 (53.6-72.0)	62.4 (54.0-72.1)	63.5 (52.3-70.0)	0.41
BMI (kg/m^2^)	23.7 (20.5-27.0)	24.0 (20.4-27.0)	23.4 (20.6-26.7)	0.75
Diagnosis of cancer, n (%)	141 (44.8)	90 (45.2)	51 (44.0)	0.90
Type of cancer				0.68
Hematological, n (%)	39 (27.7)	26 (28.9)	13 (25.5)	
Gastrointestinal, n (%)	38 (27.0)	21 (23.3)	17 (33.3)	
Prostate, n (%)	13 (9.2)	10 (11.1)	3 (5.9)	
Brain, n (%)	13 (9.2)	8 (8.9)	5 (9.8)	
Lung, n (%)	9 (6.4)	7 (7.8)	2 (3.9)	
Others/undetermined, n (%)	29 (20.6)	18 (20.0)	11 (21.6)	
COVID-19 as main diagnosis, n (%)	33 (10.5)	22 (11.1)	11 (9.5)	0.71
Hospitalization site, n (%)				0.90
ICU, n (%)	185 (58.7)	113 (56.8)	72 (62.1)	
Wards, n (%)	89 (28.3)	60 (30.2)	29 (25.0)	
Cardiac unit, n (%)	15 (4.8)	9 (4.5)	6 (5.2)	
Others, n (%)	26 (8.3)	17 (8.5)	9 (7.8)	
Laboratory				
Hemoglobin (g/dL)	9.4 (8.1-11.1)	9.7 (8.3-11.1)	9.2 (7.7-11.0)	0.35
Albumin (g/dL)	2.6±0.7	2.6±0.7	2.7±0.7	0.27
BUN (mg/dL)	25 (16-43)	24 (13-41)	28 (17-44)	0.29
Creatinine (mg/dL)	1.09 (0.70-1.91)	1.07 (0.69-1.93)	1.16 (0.71-1.87)	0.46
Sodium (mEq/L)	143±7	142±7	143±7	0.14
Chloride (mEq/L)	106±13	104±14	108±11	0.11
Phosphorus (mg/dL)	3.4 (2.6-4.5)	3.2 (2.4-4.2)	3.6 (2.6-5.3)	0.001
Potassium (mEq/L)	3.9±0.7	3.9±0.7	3.9±0.8	0.84
Magnesium (mEq/L)	1.9 (1.7-2.2)	1.9 (1.7-2.2)	1.9 (1.7-2.2)	0.22
Parenteral diet, n (%)	84 (26.7)	54 (27.1)	30 (25.9)	0.90
Calories on day 1 (kcal/kg)	7.0 (5.8-10.0)	7.0 (5.8-10.1)	6.7 (5.8-10.2)	0.40

Data are reported as number of patients (%), means±SD, or median (interquartile range). *T*-test, chi-squared test, or Mann-Whitney U test. RS: refeeding syndrome; BMI: body mass index; ICU: intensive care unit; BUN: blood urea nitrogen.

According to the adopted criteria, the incidence of RS in the studied population was 36.8%.

**Figure 2:**
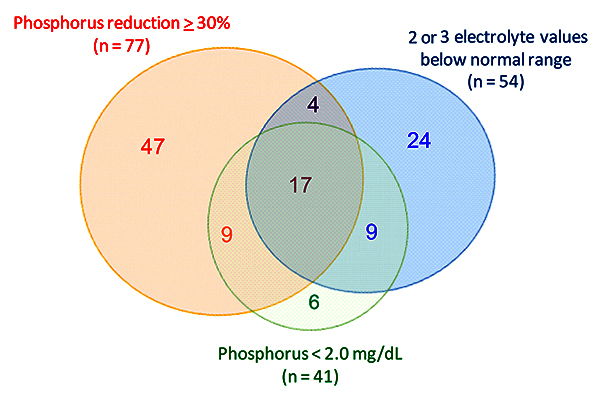
Distribution of 116 patients (36.8%) who developed refeeding syndrome according to the diagnostic criteria.

RS was diagnosed in 116 patients who received enteral or parenteral nutrition. Another 199 did not meet the diagnostic criteria. In 77, there was a reduction of 30% in basal phosphorus, 54 showed alterations of 2 or more electrolytes below normal levels, and 41 had phosphorus levels below 2.0 mg/dL (Figure 2).

**Figure 3:**
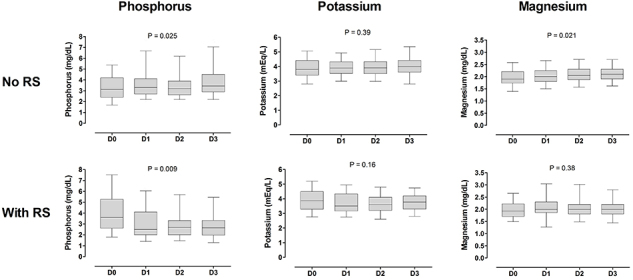
Electrolyte changes from day 0 to day 3 (D0-D3) after reintroduction of the diet in patients with and without refeeding syndrome (RS). Data are reported as medians and interquartile ranges. One-way ANOVA or Kruskal-Wallis test.

The distribution of serum P, K, and Mg levels at baseline and in the first three days of nutrition is presented in Figure 3. The analysis of the graphs shows a significant difference in phosphorus values over the three days of nutritional evaluation in the group of patients with RS (P=0.009) and without RS (P=0.025). In this period, 44.8% of patients with RS and 46.2% without RS diagnosis received some form of intravenous electrolyte repletion (P, K, or Mg, P=0.81). There was no significant difference between the proportions of patients with or without RS diagnosis who received at least one of these intravenous electrolyte repletions from baseline to day 3 (44.8 and 46.2%, respectively; P=0.81), although patients with RS diagnosis more frequently received intravenous phosphorus repletion (14.7 and 6.0%, respectively; P=0.015).

Logistic regression was used to identify risk factors associated with the development of RS (Table 2). Male sex was associated with a 48% reduction in the risk of developing RS (P=0.012), while each 1 mEq/L increase in serum sodium level increased the risk by 5% (P=0.024).

**Table 2:** Logistic regression analysis for prediction of refeeding syndrome development.

Variable	Unadjusted model			Adjusted model		
	OR	95%CI	P-value	OR	95%CI	P-value
Female	1.47	(0.93-2.33)	0.10	1.87	(1.11-3.12)	0.018
Age (years)	1.00	(0.98-1.01)	0.69	-	-	-
Diagnosis of cancer	0.92	(0.58-1.47)	0.74	-	-	-
BMI <18.5 kg/m^2^	1.80	(0.84-3.84)	0.13	2.06	(0.93-4.56)	0.076
Critical care	1.25	(0.78-1.99)	0.36	-	-	-
Serum albumin (g/dL)	1.28	(0.83-1.98)	0.27	-	-	-
Phosphorus (mg/dL)	1.20	(1.02-1.41)	0.031	1.24	(1.04-1.49)	0.018
Magnesium (mg/dL)	1.12	(0.55-2.30)	0.76	-	-	-
Potassium (mEq/L)	0.96	(0.66-1.40)	0.83	-	-	-
Creatinine (mg/dL)	0.98	(0.92-1.05)	0.58	-	-	-
BUN (mg/dL)	1.01	(0.99-1.02)	0.29	-	-	-
Sodium (mEq/L)	1.03	(0.99-1.07)	0.14	1.05	(1.01-1.10)	0.013
Parenteral diet	0.95	(0.56-1.60)	0.84	-	-	-
Calories on D1 (kcal/kg)	0.98	(0.92-1.03)	0.40	-	-	-

OR: odds ratio; CI: confidence interval; BMI: body mass index; BUN: blood urea nitrogen. D1: day 1.

**Figure 4:**
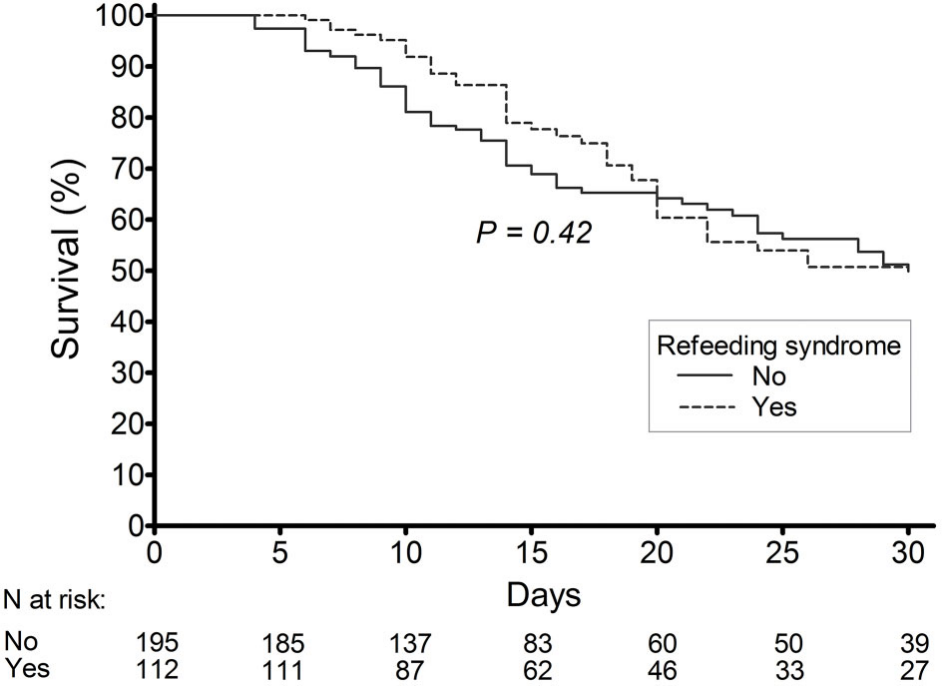
Survival analysis at 30 days by the Kaplan-Meier test.

The Kaplan-Meier test was used to assess 30-day survival of patients. No significant difference was found between the groups with and without RS (50.7 and 49.9%, respectively; P=0.42), as shown in Figure 4.

The Cox regression model was applied to predict the risk of death within 30 days after the start of feeding. Age was associated with a 2% increase in risk for each additional year of life (P=0.015). Admission to the ICU was correlated with a significant increase in risk, with a hazard ratio (HR) of 2.74 (P<0.001). Parenteral nutrition was associated with a reduced risk of death in the univariate model but lost significance after adjustment for the other variables (HR: 0.47, 95%CI: 0.20-1.09, P=0.08; Table 3).

**Table 3:** Cox regression analysis for prediction of 30-day mortality after initiation of diet.

Variable	Unadjusted model			Adjusted model		
	OR	95%CI	P-value	OR	95%CI	P-value
RS	0.86	(0.57-1.28)	0.45	0.81	(0.54-1.21)	0.30
Female	0.98	(0.66-1.43)	0.90	-	-	-
Age (years)	1.02	(1.01-1.03)	0.009	1.02	(1.01-1.03)	0.015
Cancer diagnosis	0.96	(0.65-1.41)	0.84	-	-	-
BMI <18.5 kg/m^2^	0.82	(0.38-1.76)	0.60	-	-	-
Serum albumin (g/dL)	1.14	(0.80-1.61)	0.47	-	-	-
Phosphorus (mg/dL)	1.09	(0.96-1.23)	0.22	-	-	-
Magnesium (mg/dL)	1.00	(0.99-1.02)	0.46	-	-	-
Potassium (mEq/L)	1.20	(0.82-1.75)	0.35	-	-	-
Creatinine (mg/dL)	0.99	(0.95-1.04)	0.77	-	-	-
BUN (mg/dL)	1.01	(1.01-1.02)	0.021	1.00	(0.99-1.01)	0.68
Sodium (mEq/L)	1.02	(0.99-1.05)	0.33	-	-	-
Critical care	2.44	(1.54-3.88)	< 0.001	2.74	(1.69-4.44)	<0.001
Parenteral diet	0.26	(0.13-0.54)	< 0.001	0.47	(0.20-1.09)	0.08
Calories on D1 (kcal/kg)	0.91	(0.85-0.97)	0.005	0.96	(0.89-1.04)	0.32

HR: hazard ratio; CI: confidence interval; RS: refeeding syndrome; BMI: body mass index; BUN: blood urea nitrogen.

In a sensitivity analysis, we explored the differences between the subgroup of 47 patients whose RS diagnosis was established exclusively on a phosphorus reduction of ≥30% after diet introduction and the subgroup of 69 patients with RS based on a phosphorus level <2.0 mg/dL or a drop below the normal laboratory range in at least 2 of these 3 electrolytes, including 30 patients who also fulfilled the 30% phosphorus reduction criteria (Figure 2). Baseline serum phosphorus, potassium, and magnesium levels were significantly higher in the subgroup with RS based on phosphorus reduction ≥30% only (Supplementary Table S1). As expected, phosphorus levels from day 1 to day 3 after diet introduction were significantly lower than baseline in the subgroup with RS based on the phosphorus reduction rate, whereas that change was not significant in the subgroup with RS based on phosphorus level <2.0 mg/dL or on a drop below the normal range in more than one electrolyte (Supplementary Figure S1). The subgroup with RS diagnosis based exclusively on phosphorus reduction rate less frequently received intravenous phosphate from baseline to day 3 (6.4 *vs* 20.3%, P=0.04). Other relevant differences between the subgroups were found, including higher serum creatinine (1.71 *vs* 0.98 mg/dL, P<0.001) and BUN (38 *vs* 24 mg/dL, P<0.001) among those with RS diagnosis based on phosphorus reduction rate (Supplementary Table S1. There was no significant difference in the 30-day survival between the subgroups (52.8 *vs* 47.0%, P=0.69; Supplementary Figure S2).

## Discussion

This observational study involved 315 patients previously fasting in a university hospital, who received enteral or parenteral nutrition for at least 3 consecutive days. A high incidence of RS was found based only on electrolyte changes of P, K, and Mg. The clinical profile of the patients may also help explain the high incidence of RS, as most were in the ICU when nutritional therapy was initiated. However, the development of RS was not associated with an increased risk of death within 30 days.

The literature reports a highly variable incidence of RS. In two meta-analyses, the incidence of RS ranged from 0 to 80% and from 0 to 62% (9,14). Higher percentages were observed when the diagnosis was based solely on electrolyte changes, and lower percentages when considering the combination of electrolyte disturbances and clinical symptoms. In the subgroup analysis of one meta-analysis, critically ill patients and those receiving more than 20 kcal/kg/day had rates around 44%. In contrast, Rio et al. (11), analyzing clinical and critical patients, had found an incidence of only 2%, with criteria that included, in addition to electrolyte changes, the presence of edema, fluid overload, or organ dysfunction. The incidence among hospitalized clinical patients, using electrolyte disturbances as the criterion, ranged from 8 to 18.7% (19,20). A recent study involving non-critical elderly patients who received enteral nutrition demonstrated how the application of different diagnostic methods altered the incidence of refeeding syndrome (21). The three criteria included: phosphorus drop below 2.0 mg/dL, the Friedli criteria (adopted in the present study), and the ASPEN criteria, based on percentage drops of 10 to 30% from baseline levels of phosphorus, magnesium, or potassium. The incidences were 12.9, 31.8, and 65.9%, respectively. Thus, the great heterogeneity of studies, with different definitions of RS and distinct populations, may explain the large variation in the reported incidence of RS.

In the present study, no significant difference was found in baseline characteristics between groups that did or did not develop RS, regarding sex, age, weight, BMI, cancer diagnosis, presence of COVID-19, location of hospitalization, and laboratory data, except for the baseline phosphorus values, which were higher in patients who developed RS.

In patients with RS, serum phosphorus levels showed a significant decrease in the first three days after the introduction of the diet compared to values on day 0. This was expected, since a drop of 30% or more was one of the diagnostic criteria for RS. The current understanding is that there is a body deficit not only of phosphorus but also of potassium and magnesium in the development of RS (2,7). However, in our study, there were no significant drops in potassium or magnesium in patients who developed RS, while a slight increase in magnesium levels was observed in patients who did not have RS. The absence of a drop in potassium and magnesium in patients who developed RS could be attributed to the rapid prescription and replacement of these electrolytes.

No significant difference in the incidence of RS between the use of enteral or parenteral nutrition was detected in our study. In the literature, some observed a higher incidence with enteral nutrition (15), whereas others found a higher incidence with exclusive parenteral nutrition, especially with high initial caloric loads and the ready-to-use parenteral nutrition (22,23).

Concerning the predictors for the development of RS, only higher serum phosphorus and sodium levels before the start of the diet and female gender were identified as risk factors. Adika et al. (24), in evaluating the ASPEN guidelines for the diagnosis of RS, considered that higher pre-feeding phosphorus levels have six times the risk of meeting the criteria for severe RS. This is because it is more difficult to reduce serum phosphorus levels when they are already very low before reintroducing feeding. As for sodium and female gender, we did not find their mention as risk factors in previous studies, perhaps linked to the specific characteristics of our sample.

We did not observe a significant association of RS with other variables previously described as risk factors, such as a higher initial caloric load, as noted by some authors (25,26), low BMI (13), older age (27), or being a critically ill patient (10,28-30). This lack of association could have occurred due to the early identification by MNTT of those at higher risk of developing RS, such as the elderly and patients with low BMI, acting preventively. The caloric quotas prescribed by the team would be more liberal for low-risk patients and more restrictive for those at high or very high risk.

In a sensitivity analysis, we found that patients with RS diagnosed exclusively by a reduction in serum phosphorus of 30% or more had a baseline phosphorus significantly higher than those with RS diagnosed by low absolute phosphorus levels or more than one electrolyte below the laboratory normal range after diet introduction. The reasons for a higher baseline serum phosphorus in the first subgroup deserve further discussion. It is plausible that some were selected due to “regression toward the mean” phenomenon, i.e., they could have fulfilled the criterion of a 30% reduction in serum phosphorus due to transient day-to-day variations in serum levels, with values randomly higher at baseline and lower at some point after diet introduction.

However, considering that the subgroup of patients with RS diagnosis based exclusively on phosphorus reduction rate also had higher baseline serum creatinine and BUN levels, we cannot rule out a lower glomerular filtration rate as a cause of phosphorus retention and increased serum levels. We did not estimate glomerular filtration rate using equations based on serum creatinine, adjusted for sex and age, because such equations are not validated for inpatients in unstable clinical conditions (31).

On the other hand, serum phosphorus levels did not significantly change after the diet introduction in the subgroup of patients with RS diagnosis based on the low absolute phosphorus levels or the presence of more than one electrolyte below the laboratory normal range. We believe that earlier and more frequent intravenous electrolyte repletion in that subgroup could have mitigated phosphorus decline after diet initiation. The number of patients with RS diagnosis based on phosphorus below 2.0 mg/dL receiving intravenous phosphate at any moment from baseline to day 3 was lower than expected for a group with such a laboratory alteration. It is plausible that other patients were appropriately diagnosed and repleted with phosphate after the third day of the diet, but no extended period was analyzed.

In our study, the development of RS was not associated with an increased risk of death and, unsurprisingly, older age and admission to the ICU were predictors of 30-day mortality. Friedli et al. (32) found an increase in 180-day mortality in non-critical malnourished patients diagnosed using the same criteria we employed. However, other authors did not identify a difference in mortality between the groups with and without RS (21,33). A meta-analysis including 13 studies has shown a trend for increased mortality at 30 days and a significant increase at 6 months of follow-up (34). The interesting finding of this meta-analysis was the relationship between increase in mortality rate and lack of prescription and follow-up of patients by an MNTT. This factor may explain the absence of association between 30-day mortality and RS in our results, with all patients being followed up by the MNTT.

Two studies found that low initial caloric rate (<20 kcal/kg/day), compared to high rates (>20 kcal/kg/day), reduced the risk of mortality at 60 days (25) and 6 months (26). However, a systematic review found that high caloric intake is not necessarily associated to unfavorable outcomes, provided that clinical and electrolyte monitoring is done daily (8). Mortality in RS seems to be more strongly associated with the progressive increase in the risk of developing RS than with exposure to different caloric loads (35). In our sample, no initial caloric load greater than 20 kcal/kg/day was prescribed, and for patients considered at high risk, the proposal was to start within the range of 5 to 15 kcal/kg/day.

Although this study presents some important findings about RS in our studied population, it had some limitations. First, its retrospective nature led to recording failures and a significant loss of participants due to missing data. The absence of laboratory data led to the exclusion of nearly half of the participants who received enteral or parenteral nutrition by the third day. Second, there was a lack of reliable data on fasting time and weight loss before the start of enteral or parenteral nutritional therapy. This prevented the inclusion of these variables in the analysis of risk factors for RS development. BMI, collected only cross-sectionally in all patients, does not adequately reflect nutritional status, even though most patients were admitted malnourished and some had a long history of inadequate intake. A third limitation related to the intervention of the MNTT in the indication of replacement of electrolytic deficits and in the reduction of caloric intake for patients deemed at risk, which may have interfered with the final incidence of RS and even mortality. Another limitation would be that our sample may not necessarily reflect the final incidence in other hospitals of the same size. Most patients were oncological and hematological, as this hospital is a reference center in these specialties in a large area of the State of Rio de Janeiro. On the other hand, the study also had strengths, such as the considerable sample size and prospective data collection in a specific spreadsheet by the MNTT.

In conclusion, a high incidence of RS was found in the studied population. The identified risk factors were being female and having higher serum phosphorus and sodium levels before the initiation of enteral or parenteral diet. However, the presence of RS was not associated with risk of death within 30 days, likely due to early diagnosis and rapid intervention to correct electrolyte deficits.

## Data Availability

All data generated or analyzed during this study are included in this published article.

## Supplementary Materials

Click to view [pdf].
