# Supplementary material for: Incidence and risk factors of refeeding syndrome in inpatients undergoing enteral and parenteral nutrition hospitalized in a university medical hospital
Source: Braz J Med Biol Res. 2026 Aug 17;59:e15114. doi: 10.1590/1414-431X2026e15114 (PMC13480946; doi:10.1590/1414-431X2026e15114)
Supplement: Supplementary file 1 [file 1414-431X-bjmbr-59-e15114-suppl.pdf]

Figure S1. Serum phosphorus changes from day 0 to day 3 after reintroduction of the diet according to the refeeding syndrome (RS) diagnostic criteria.

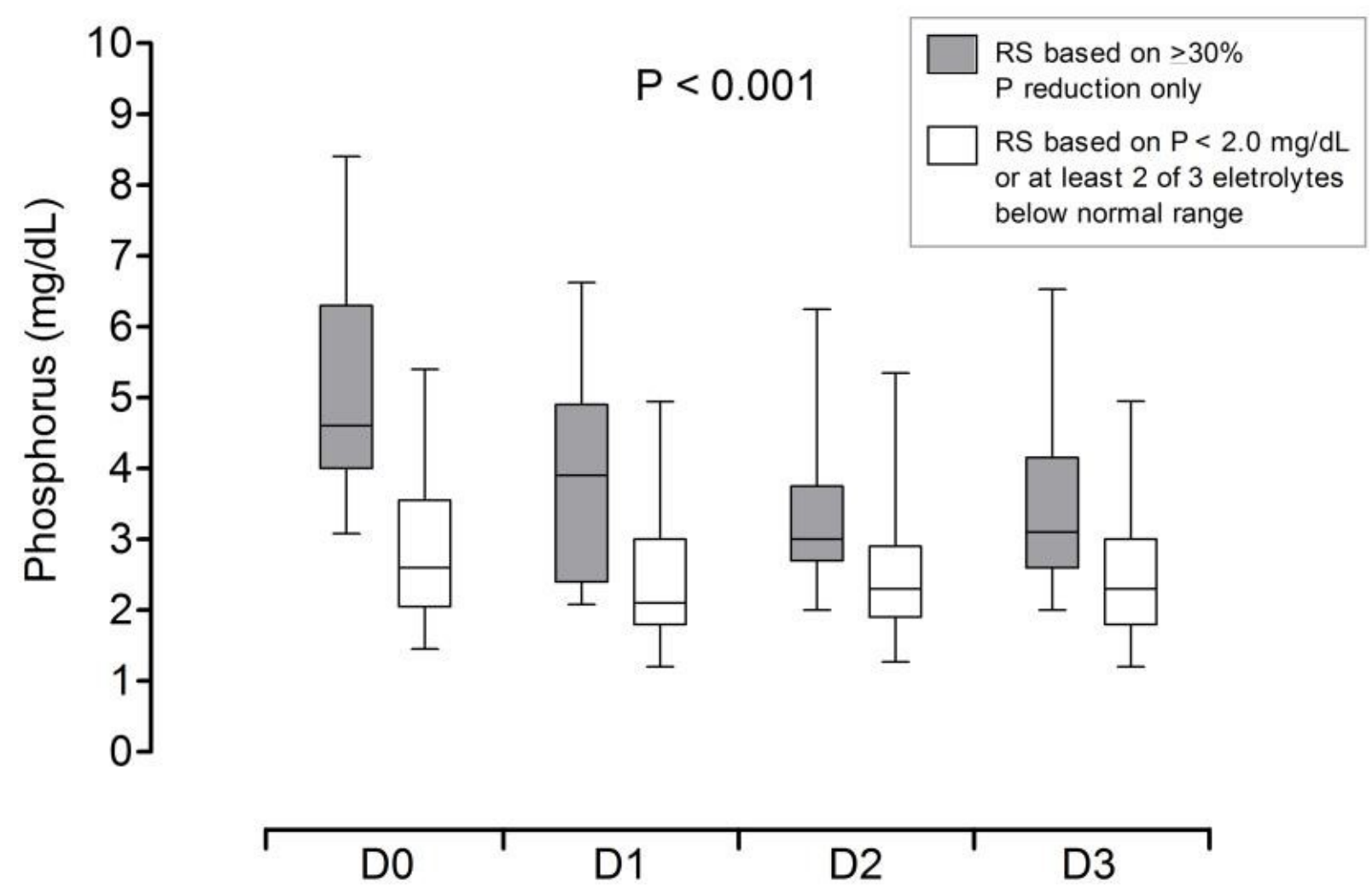

Figure S2. Survival analysis at 30 days according to according to the refeeding syndrome (RS) diagnostic criteria.

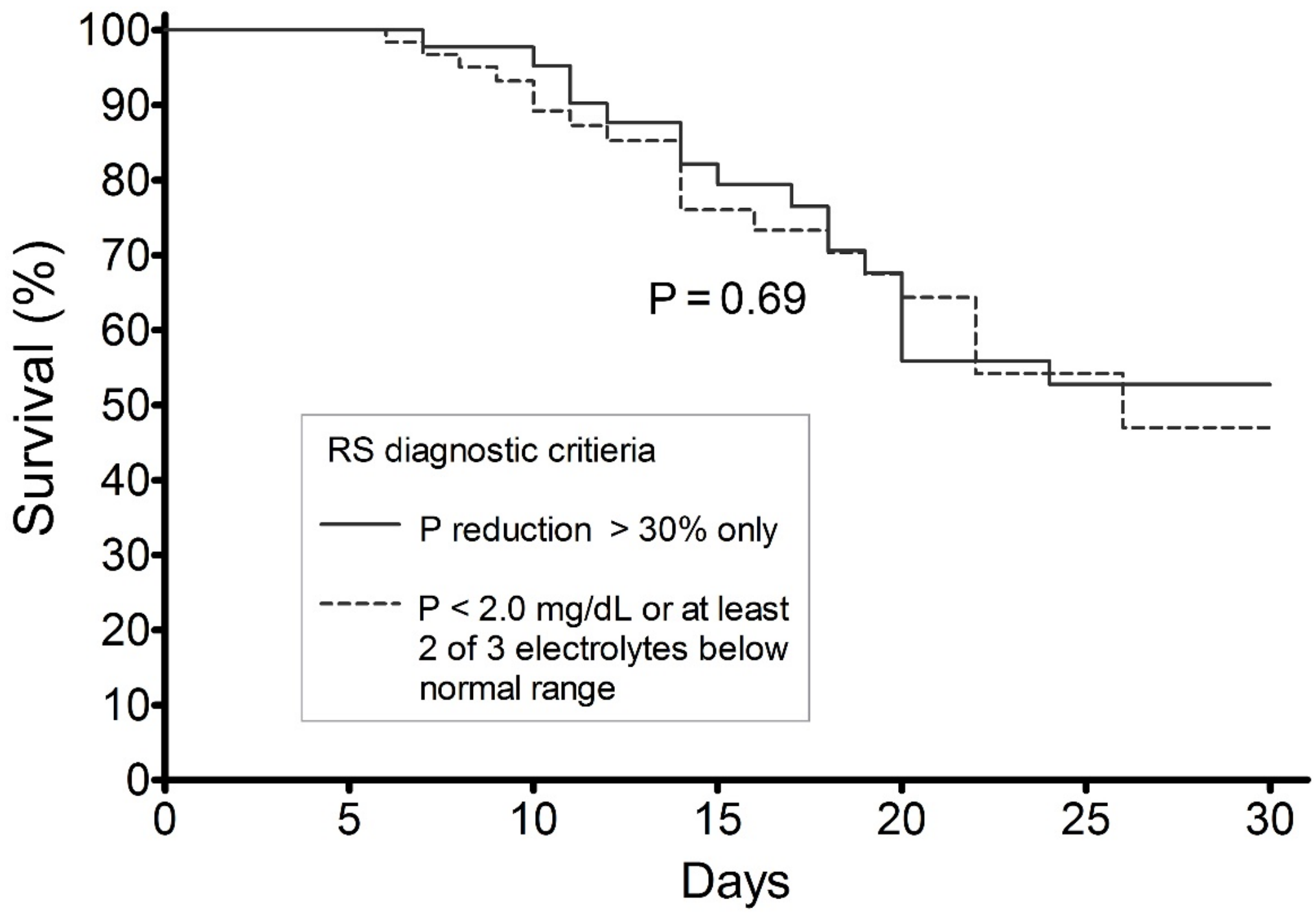

Table S1. Main characteristics of patients at baseline according to the RS diagnostic criteria.

	RS diagnostic criteria		P-value
	P reduction ≥30% (n=47)	P<2.0 mg/dL or a drop below the normal range in at least 2 of 3 electrolytes (n=69)	
Female, n (%)	23 (48.9)	36 (52.2)	0.85
Age (years)	60 ± 13	63 ± 15	0.21
Weight (kg)	65 (60–72)	62 (50–70)	0.58
BMI (kg/m^2)	24.4 (21.8–25.8)	23.0 (19.9–27.1)	0.66
Diagnosis of cancer, n (%)	18 (38.3)	33 (47.8)	0.34
ICU admission, n (%)	36 (76.6)	36 (52.2)	**0.011**
Laboratory			
Hemoglobin (g/dL)	9.2 (7.8–11.7)	8.8 (7.7–10.9)	0.26
Albumin (g/dL)	2.6 ± 0.7	2.8 ± 0.7	0.30
BUN (mg/dL)	38 (24–48)	24 (14–37)	**<0.001**
Creatinine (mg/dL)	1.71 (1.01–2.61)	0.98 (0.60–1.40)	**<0.001**
Sodium (mEq/L)	143 ± 7	143 ± 6	0.58
Chloride (mEq/L)	108 ± 15	108 ± 8	0.89
Phosphorus (mg/dL)	4.6 (4.0–6.3)	2.6 (2.1–3.6)	**<0.001**
Potassium (mEq/L)	4.3 ± 0.7	3.6 ± 0.7	**<0.001**
Magnesium (mg/dL)	2.1 ± 0.4	1.9 ± 0.4	**0.006**
Parenteral diet, n (%)	7 (14.9)	23 (33.3)	**0.031**
Calories in day 1 (kcal/kg)	6.4 (5.6–8.3)	7.4 (5.9–11.4)	0.07

Frequency data are reported as number of patients (%), mean ± standard deviation or median (interquartile range). RS, refeeding syndrome; BMI, body mass index; ICU, intensive care unit; BUN, blood urea nitrogen. Bold type indicates statistically significant.
